# Vibration Training Triggers Brown Adipocyte Relative Protein Expression in Rat White Adipose Tissue

**DOI:** 10.1155/2015/919401

**Published:** 2015-06-01

**Authors:** Chao Sun, Ruixia Zeng, Ge Cao, Zhibang Song, Yibo Zhang, Chang Liu

**Affiliations:** ^1^Department of Endocrinology, The First Affiliated Hospital of Liaoning Medical University, Jinzhou 121001, China; ^2^Department of Anatomy, Liaoning Medical University, Jinzhou 121001, China; ^3^Department of Pathogeny Biology, Liaoning Medical University, Jinzhou 121001, China

## Abstract

Recently, vibration training is considered as a novel strategy of weight loss; however, its mechanisms are still unclear. In this study, normal or high-fat diet-induced rats were trained by whole body vibration for 8 weeks. We observed that the body weight and fat metabolism index, blood glucose, triglyceride, cholesterol, and free fatty acid in obesity rats decreased significantly compared with nonvibration group (*n* = 6). Although intrascapular BAT weight did not change significantly, vibration enhanced ATP reduction and increased protein level of the key molecule of brown adipose tissue (BAT), PGC-1*α*, and UCP1 in BAT. Interestingly, the adipocytes in retroperitoneal white adipose tissue (WAT) became smaller due to vibration exercise and had higher protein level of the key molecule of brown adipose tissue (BAT), PGC-1*α*, and UCP1 and inflammatory relative proteins, IL-6 and TNF*α*. Simultaneously, ATP content and PPAR*γ* protein level in WAT became less in rats compared with nonvibration group. The results indicated that vibration training changed lipid metabolism in rats and promoted brown fat-like change in white adipose tissues through triggering BAT associated gene expression, inflammatory reflect, and reducing energy reserve.

## 1. Introduction

Inactive life style in China is increasingly inducing obesity and relative diseases, such as diabetes. Therefore, combating the obesity and diabetes epidemic is urgently required. So far, due to limited efficacy or side effects, many pharmacological weight loss therapies, aiming at reducing energy intake, have been hampered. Accordingly, exercise still is considered as safe and efficient method. However, special groups of people, who are disable to move as a result of deformity on a person or some diseases, need a novel exercise method.

Whole body vibration (WBV) exercise is an emerging training method as complement to programs aimed at weight loss in overweight people. The addition of whole body vibration to both static and dynamic exercises appears to significantly increase oxygen uptake in overweight women [1]. Whole body vibration is reported to stimulate the secretion of growth hormone (GH) and testosterone in male students [2]. Some research supported that vibration training may play a significant role in preventing osteoporosis and losing weight [3–5].

Brown adipose tissue (BAT) plays a role in the regulation of energy balance and maintenance of body weight in rodents. BAT is able to generate heat, adjust the body temperature, and participate in energy consumption [6]. PPAR*γ* coactivator *α* (PGC-1*α*) and UCP1 play an important role in BAT [7, 8]. PGC-1*α* is a key mitochondrial-related transcription factor that mediates coactivation of key nuclear hormone receptor-dependent gene transcription as well as mitochondria biogenesis [9]. UCP1 makes the respiratory chain and ATP synthesis of decoupling, thus affecting heat production and energy metabolism [10, 11]. Recently, brown-like adipocytes that appear in white fat depots have been called “brite” (from brown-in-white) or “beige” adipocytes and have characteristics similar to brown adipocytes, in particular, the capacity for uncoupled respiration [12].

Exercise had been considered as a new physiological stimulus for brown adipose tissue activity [13]. As a special exercise, in this study, the WBV exercise reduced the body weight and blood glucose, triglyceride, cholesterol, and free fatty acid in obese rats and increased key molecule of brown adipose tissue PGC-1*α* and UCP1 expression in rat WAT. It indicated that WBV exercise may turn bad “fat” WAT into good “fat” BAT.

## 2. Materials and Methods

### 2.1. Animals

Male Sprague-Dawley rats (8 weeks old, 180–200 g) were purchased from Liaoning Laboratory Animals Center and housed one per cage in a controlled temperature (22°C) and humidity (55–65%) room under a light-dark cycle (lights on at 9:00 am and off at 21:00 pm). 24 rats were divided into 2 groups after feeding for a week in the above conditions. High-fat diet group (*n* = 12) was lavaged by 2 mL/kg high-fat emulsion (cholesterol 100 g/L, lard 200 g/L, and sodium cholate 20 g/L), once daily for eight weeks, and fed with normal chow. Normal diet group (*n* = 12) was fed with normal chow (320 kcal/100 g/day). After feeding for eight weeks and randomly grouping, half of the rats in high-fat diet group and normal diet group were trained for 15 min twice a day and intermittent for 5 min in the frequency 25 Hz in LD-P vertical vibration machine (Huanzhen Machinery Limited Company, Guangdong, China). Vibration intervention carried out from Monday to Saturday, rested on Sunday.

After 8 weeks of the training program, rats from all groups were weighed after 12 h fasting and anesthetized by i.p. injection of chloral hydrate (40 mg/100 g). Cervical artery blood samples were collected in heparin tubes, and plasma was stored at −80°C. Retroperitoneal WAT and intrascapular BAT were excised, weighed, and frozen at −80°C until analysis.

The experimental procedures followed the Guiding Principles for the Care and Use of Animals in the Waseda University Institutional Animal Care and Use Committee (approved number: 2011-A19).

### 2.2. Methods

#### 2.2.1. Measurements of Blood and Tissue Parameters

ELISA kits (Nanjing Jiancheng Bioengineering Institute, Nanjing, China) were used to determine triglycerides, free fatty acid, and cholesterol levels in rat sera and ATP content in WAT and BAT.

#### 2.2.2. H&E Staining and Cell Size Quantitation

Standard H&E staining was performed on 5 *μ*m paraffin sections of WAT and interscapular brown adipose tissue. Cell diameter was measured in the H&E-stained sections of three individual samples in each group using Image J.

#### 2.2.3. Immunohistochemistry

Immunohistochemical staining was applied to paraffin sections of rat retroperitoneal WAT to examine expression of PGC-1*α* and UCP1. The 5 *μ*m serial sections were incubated in 4% paraformaldehyde for 12 hours at 4°C. Endogenous peroxidase was inactivated with 1% hydrogen peroxide in methanol for 15 min at 4°C. PGC-1*α* (1 : 200 dilution) and UCP1 (1 : 200 dilution) primary antibodies were added in 1% BSA solution and sections were incubated overnight at 4°C. Secondary anti-rabbit antibody was added in PBS buffer with normal mouse serum for 30 min at room temperature. Proteins were visualized using the Vectastain Elite ABC Kit (Burlingame, CA) for 30 min at room temperature and further incubation was carried out with diaminobenzidine (DAB) chromogen. The results were measured by Image-Pro Plus 5.02 (Media Cybemetics, USA).

#### 2.2.4. Western Blotting

Proteins in the adipose fractions obtained by the gel filtration were separated by sodium dodecyl sulfate-polyacrylamide gel electrophoresis. Samples from each fraction were mixed with an equal volume of 0.125 mol/L Tris-HCl (pH 6.8), 4% sodium dodecyl sulfate, 10% sucrose, and 0.004% bromophenol blue. These mixtures were boiled for 3 minutes in the presence of 2-mercaptoethanol for reducing conditions or incubated for 30 minutes at room temperature in the absence of 2-mercaptoethanol for nonreducing conditions. After electrophoresis, the proteins on the gel were transferred to a polyvinylidene difluoride membrane. The membrane was incubated with Block Ace and then with rabbit anti-rat PGC-1*α* (bs-1832R), PPAR*γ* (bs-4590R), UCP1 (bs-1925R), IL-6 (bs-0782R), and TNF-*α* (bs-2081R) antibody (Beijing Biosynthesis Biotechnology Company, Beijing, China). After incubation, the membrane was treated with biotin-conjugated anti-rabbit IgG antibody and then with avidin-HRP. Finally, the membrane was soaked in ECL reagent and exposed to lumino imaging analyzer FAS-1100 (Toyobo Corp., Osaka, Japan).

### 2.3. Statistical Analysis

Data are expressed as mean ± SEM and the Statistical Package for the Social Sciences (SPSS17.0) was used to perform all comparisons. Analysis of variance (ANOVA) was used to evaluate the effects of vibration training between the groups (determined by SNK test). A *P* value of less than 0.05 was considered significant for the differences.

## 3. Results 

### 3.1. Vibration Training Affects Lipid Metabolism in Obese Rats, Not Normal Rats

**Figure 1:**
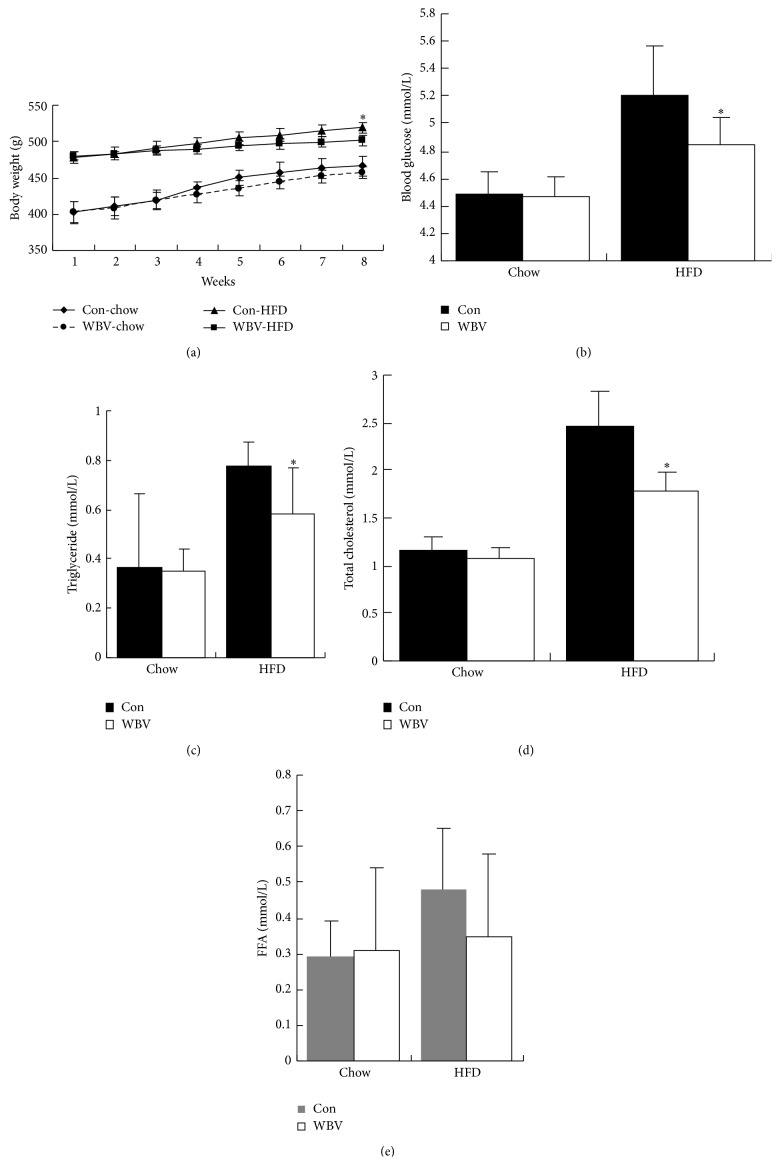
Body weight and fat metabolism index in rats treated with whole body vibration. After eight weeks of vibration training with 25 Hz, body weight in every week (a), the blood glucose (b), triglyceride (c), total cholesterol (d), and free fatty acid (FFA) (e) were compared between control and WBV groups in normal chow diet or high-fat diet group. Data are expressed as means ± SEM (*n* = 6). ^∗^ *P* < 0.05 versus control group.

To evaluate the role of vibration in obese reduction, we established rat obesity model through lavaging high-fat emulsion for eight weeks. In this study, we observed that high-fat diet rats' body weight in WBV group was lower than control group on 8th week (*P* < 0.05). There are no significant differences between control group and WBV group in high-fat diet group or not normal diet group (Figure 1(a)). It indicated vibration training reduced the weight of high-fat diet-induced obese rats but not normal diet rats after 8 weeks. Interestingly, the index of lipid metabolism in high-fat diet-induced rats, blood glucose (Figure 1(b)), triglyceride (Figure 1(c)), and total cholesterol (Figure 1(d)) was lower than that in control group (*P* < 0.05) after vibration exercise. However, compared with control group, the decrease of the above index in high-fat diet group is not significant in normal diet group (*P* > 0.05). Moreover, decreased free fatty acid levels were nonsignificant among the groups (*P* > 0.05) (Figure 1(e)). It suggested that weight loss induced by vibration training was associated with lipid metabolism in high-fat diet-induced rats. Collectively, these findings indicated that body weight loss in WBV group was dependent on the loss of adipocyte tissue, either WAT or BAT, and lipid metabolism which was influenced by vibration exercise.

### 3.2. Energy Metabolism in Intrascapular BAT of WBV-Treated Rats

**Figure 2:**
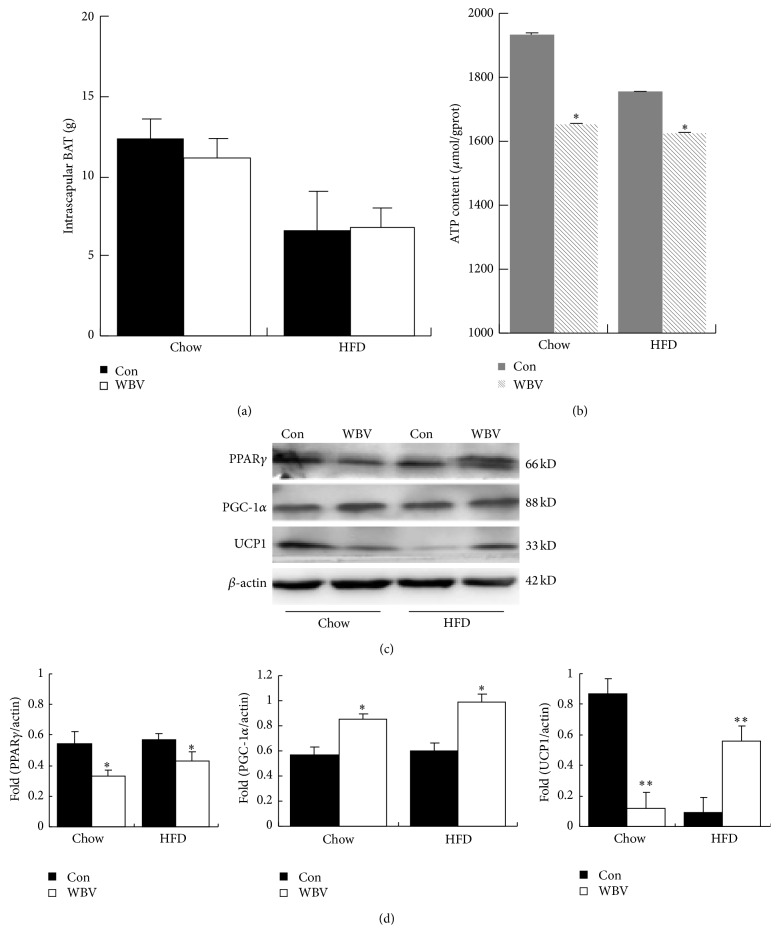
ATP content and BAT associated protein level in intrascapular BAT. (a) The weight of BAT in the different groups (*n* = 6). (b) ATP content in rat BAT (*n* = 6). (c) Western blot analysis of UCP1, PGC-1*α*, and PPAR*γ* in rat BAT. (d) UCP1, PGC-1*α*, and PPAR*γ* protein content is normalized to *β*-actin protein content. Values are means ± SEM; *n* = 3. ^∗^ *P* < 0.05 and ^∗∗^ *P* < 0.01 versus control group.

BAT is important to produce heat by energy metabolism in adipose tissue. The weight of intrascapular BAT was not influenced by WBV exercise (Figure 2(a)). UCP1, PGC-1*α*, and PPAR*γ* protein expression, which were associated with energy metabolism in the BAT, were analysed by western blotting. Quantitative data of UCP1, PGC-1*α*, and PPAR*γ* protein showed that WBV led to decreased protein levels of PPAR*γ*, higher protein levels of PGC-1*α* in both control and HFD groups, and increased protein levels of UCP1 in HFD groups (Figures 2(c) and 2(d)). However, ATP content in BAT decreased more in normal control than that in obese groups when compared with WAT (Figure 2(b)). It is important to note that protein levels of UCP1 decreased significantly after WBV when ATP content in BAT became less. It indicated that UCP1 was a master regulator of WBV-induced energy metabolism in BAT, especially in normal rats.

### 3.3. Brown Adipocyte-Like Change in WAT of Obese Rats

**Figure 3:**
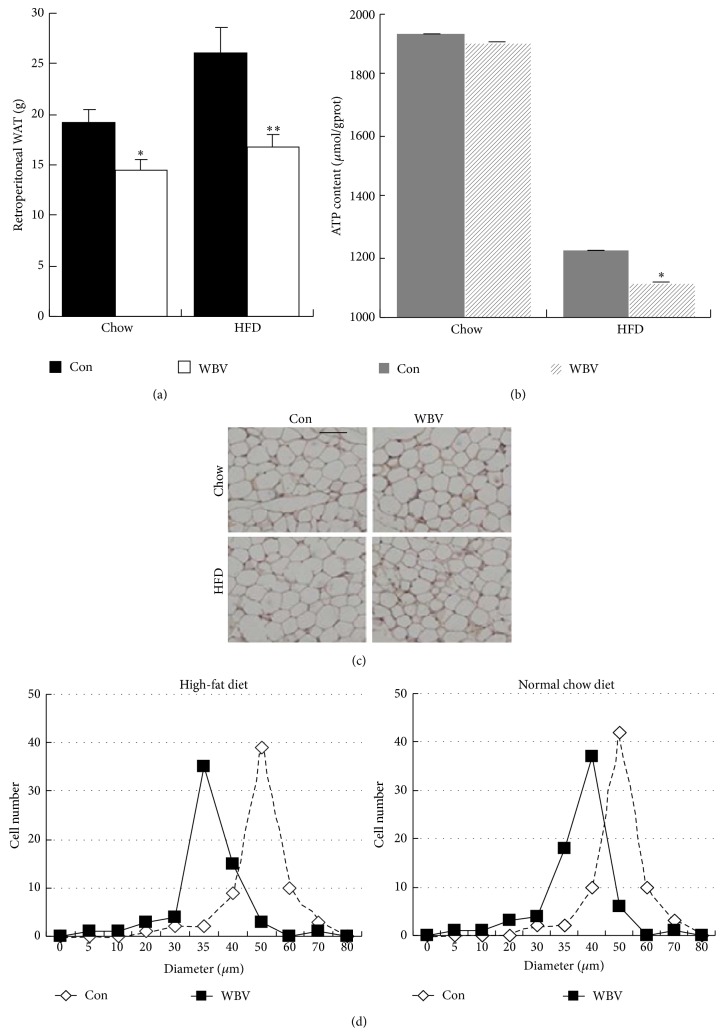
Morphology change in retroperitoneal WAT. (a) The weight of BAT in the different groups (*n* = 6). (b) ATP content in rat BAT (*n* = 6). (c) H&E staining of WAT (scale bar = 40 *μ*m, magnification, ×400). (d) Quantification of adipocyte diameter of retroperitoneal WAT from rats in the different groups (data were collected from H&E-stained sections from three individual rats, five fields per rat, and 10–15 cells per field in each group, using Image J software). Data was expressed as mean ± SEM. ^∗^ *P* < 0.05 versus control group.

**Figure 4:**
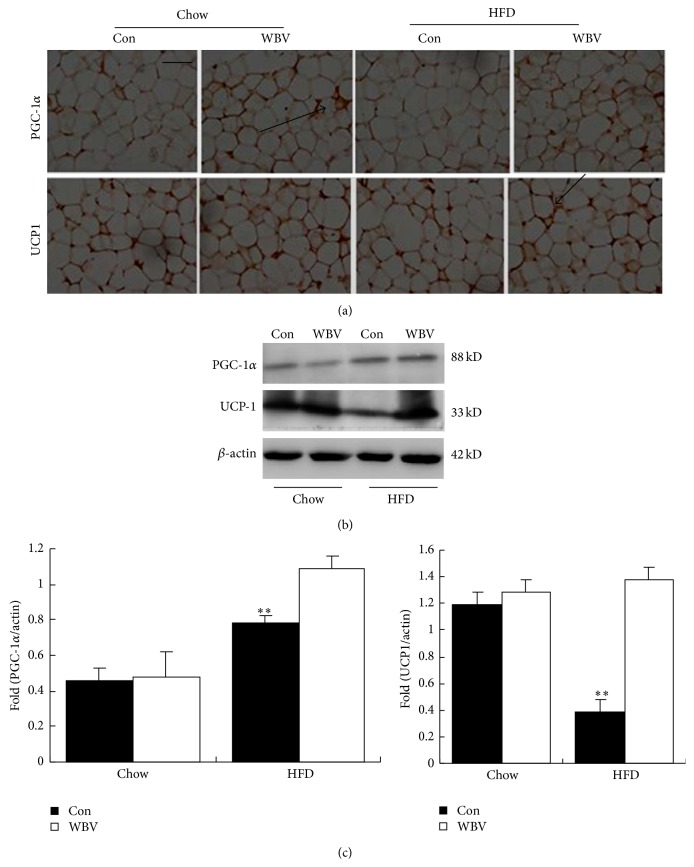
Protein expression of UCP1 and PGC-1*α* in WAT. (a) UCP1 and PGC-1*α* were detected by immunohistochemistry. Single UCP1 and PGC-1*α* immunoreactive adipocyte were closely associated with brown adipocytes (arrows). Scale bar: 40 *μ*m, magnification, ×400. (b) Western blot analysis of UCP1 and PGC-1*α* in rat BAT. (c) UCP1 and PGC-1*α* protein content is normalized to *β*-actin protein content. Values are means ± SEM; *n* = 3. ^∗^ *P* < 0.05 and ^∗∗^ *P* < 0.01 versus control group.

As shown in Figure 3(a), retroperitoneal WAT weight in WBV group was less than that in control group, either in HFD group (*P* < 0.01) or in normal group (*P* < 0.05). Accordingly, to test whether WBV stimulation is followed with mitochondrial uncoupling, we measured ATP content in fat tissue. It showed that ATP content reduced obviously in HFD groups (*P* < 0.05) and did not change in normal chow groups (Figure 3(b)). In morphology, the size of adipocytes after vibration exercise became smaller than that in high-fat diet group (Figure 3(d)) and had more nucleated cell especially in HFD group (Figure 3(c)). Based on the above observations, we hypothesized that the decreased lipid deposition of the adipocyte might be a consequence of nonshivering thermogenesis in WAT. Accordingly, to test whether WBV stimulation is followed with mitochondrial uncoupling in WAT, we measured the UCP1 and PGC-1*α* protein level, which is associated with mitochondrial respiratory. In line with our hypothesis, WBV exercise significantly reduced energy reserve (Figure 3(b)), followed with increased UCP1 and PGC-1*α* protein level in HFD groups (Figures 4(a) and 4(b)). Importantly, the main effector of adaptative thermogenesis, UCP1 molecule, which characterizes the WAT brown adipocyte-like cells, increased significantly after WBV exercise (Figure 4(c)). Of note, WBV-induced increased UCP1 protein in HFD groups was distinctly more than that in normal chow groups. We conclude that WBV promoted WAT brown adipocyte-like change through increased associated brown tissue protein especially in WAT of obese rats.

### 3.4. High Expression of Proinflammatory in WBV-Treated WAT

The protein level of PPAR*γ* in HFD rats, which was key factor of adipose cell differentiation, was lower than that in control group due to vibration (*P* < 0.01) (Figures 4(a) and 4(b)). Simultaneously, the protein level of proinflammatory cytokines TNF-*α* in both HFD (*P* < 0.05) and normal chow rats (*P* < 0.01) was higher than that in control group. In addition, IL-6 protein level increased significantly in HFD rats WBV-treated WAT. Adipose cell differentiation and inflammatory factor could be involved in which WBV promoted WAT brown adipocyte-like change.

## 4. Discussion

As known, appropriate exercise training is a benefit to metabolic disorders such as obesity and diabetes [14, 15]. Recently, short-term WBV training (6 weeks) may benefit arterial function and muscle strength in deconditioned individuals who cannot perform conventional exercise in young overweight/obese normotensive women [3]. In this study, we trained the high-fat diet-induced obese rats for 8 weeks on 25 Hz vertical vibration machine. We observed that long-termed training enhanced weight loss in obese rat but not normal control rats. Importantly, the lipid metabolism indexes, such as blood glucose, triglyceride, total cholesterol, and FFA, were significantly decreased in obese rats after vibration. The same effect of WBV was reported in male C57BL/6 HFD-induced obese mice [11].

**Figure 5:**
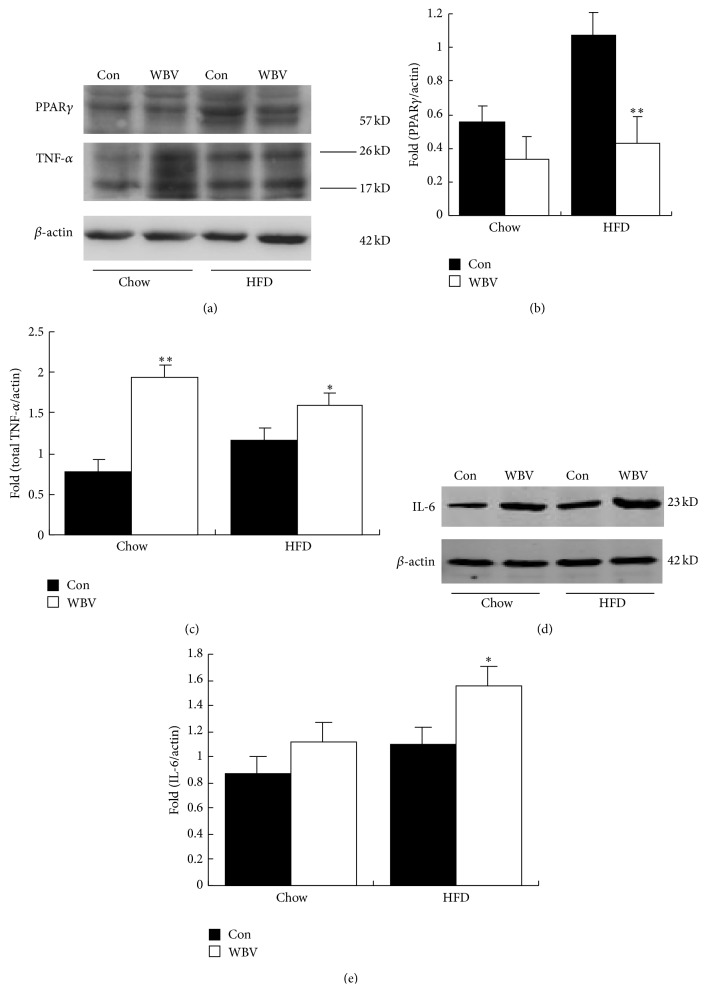
Protein expression of PPAR*γ*, IL-6, and TNF-*α* in WAT after vibration for 8 weeks. (a) Western blot analysis of PPAR*γ* and TNF-*α* in rat BAT. (b) PPAR*γ* protein content is normalized to *β*-actin protein content. (c) Total TNF-*α* protein content (26 KD and 17 KD) is normalized to *β*-actin protein content. (d) Western blot analysis of IL-6 in rat BAT. (e) IL-6 protein content is normalized to *β*-actin protein content. Values are means ± SEM; *n* = 3. ^∗^ *P* < 0.05 and ^∗∗^ *P* < 0.01 versus control group.

There are two types of adipose tissues in our body; they are white adipose tissue and brown adipose tissue. BAT is used to generate heat, can regulate our temperature, participates in energy consumption, and is relevant to keeping the body weight and WAT mainly through triglycerides to store energy. Inflammatory response induced by some stimuli may promote energy consumption and inhibit fat cell differentiation and then enhance WAT browning [16, 17]. In addition, chronic inflammation in adipose tissue has been considered as a key underlying mechanism for the development of obesity-related metabolic syndrome [17]. WBV, as a continuous stimulus, inhibited PPAR*γ* expression and enhanced IL-6 and TNF-*α* expression in WAT (Figure 5). IL-6 is produced by contracting skeletal muscles during exercise [18] and is beneficial to health effects of exercise [19]. In addition, IL-6 is required for a full induction of UCP1 protein expression in response to cold exposure and influences the UCP1 protein content WAT of both untrained and exercise trained animals [20]. On the contrary, TNF-*α* may induce endothelial cell apoptosis in brown adipose tissue of rats [21] and downregulation of UCP1 mRNA expression [22].

WAT from certain depots, in response to appropriate stimuli, can undergo a process known as browning where it takes on characteristics of BAT, notably the induction of UCP1 (uncoupling protein 1) expression and the presence of multilocular small lipid droplets [23]. Our study showed that vibration increased UCP1 protein in WAT followed with high expression of PCC-1*α* (Figures 4(b) and 4(c)) and reduced the ATP content in adipose tissue (Figure 3(b)). Uncoupling proteins (uncoupling proteins, UCPs) can change the proton electrochemical gradient and make the respiratory chain and ATP synthesis uncoupling, thus affecting heat production and energy metabolism [10, 24, 25]. Brown fat tissue specific gene UCP1 can make the mitochondrial respiratory chain oxide uncoupling [26] and lead to energy in the form of heat dissipation, to maintain body temperature and energy in steady state, so UCP1 plays an important role in the control body mass and adipose content [25]. PGC-1*α* is the strong promoter for mitochondria biosynthesis and oxidative metabolism and also can induce UCP1 higher expression in brown adipocytes. Recently, research shows that exercise can make the UCP1 protein increase both in white and brown adipose tissue and relies on the PGC-1*α* protein expression level [27]. Collectively, WBV reduced energy reserve and enhanced the process of turning WAT to BAT in obese rats.

In addition, the data indicated that UCP1 was a master regulator of WBV-induced energy metabolism in BAT in normal rats in consideration of UCP1 protein reduction and decreased ATP content (Figures 2(b) and 2(c)). It is possible that WBV had little or no effect in lipid metabolism in normal rats because of the effect of WBV on respiratory gas exchange [1] or muscle strength [4] in overweight and obese women. Certainly, the mechanism of WBV affecting lipid metabolism still needs to probe in our future studies.

## 5. Conclusions 

WBV training is a novel exercise strategy for weight loss or controlling metabolic diseases. In this study, WBV training significantly decreased body weight, fat metabolism index, blood glucose, triglyceride, cholesterol, and free fatty acid in obese rats and increased the expression of proinflammatory cytokines, IL-6 and TNF-*α*, in WAT. Moreover, WBV training affected the energy metabolism of white fat tissues and enhanced the protein expression of the brown fat tissue specific gene PGC-1*α* and UCP1 in white adipose tissue. We concluded that WBV reduced energy reserve and enhanced the process of turning WAT to BAT in obese rats.
